# 5,7-Dihydro­dibenzo[*c*,*e*]thiepine

**DOI:** 10.1107/S1600536809008769

**Published:** 2009-03-14

**Authors:** Nobuto Yoshinari, Takumi Konno

**Affiliations:** a Department of Chemistry, Graduate School of Science, Osaka University, Toyonaka, Osaka 560-0043, Japan

## Abstract

In the title compound, C_14_H_12_S, the central seven-membered C_6_S ring has a twist-boat conformation. The dihedral angle between the two benzene rings is 52.4 (1)°.

## Related literature

For the preparation of a pair of atrop diastereomeric Rh^III^ complexes having a 2,2′-bis­(2-amino­ethyl­thio­meth­yl)-1,1′-biphenyl ligand, see: Yoshinari & Konno (2008 ▶). For the synthesis, see: Foubelo *et al.* (2005 ▶).
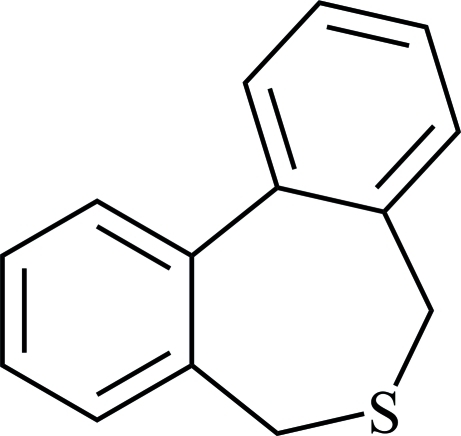


## Experimental

### 

#### Crystal data

C_14_H_12_S
*M* *_r_* = 212.30
Monoclinic, 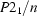

*a* = 5.645 (3) Å
*b* = 17.316 (9) Å
*c* = 11.398 (5) Å
β = 92.444 (19)°
*V* = 1113.1 (10) Å^3^
*Z* = 4
Mo *K*α radiation
μ = 0.25 mm^−1^
*T* = 200 K
0.15 × 0.15 × 0.10 mm

#### Data collection

Rigaku R-AXIS RAPID diffractometer
Absorption correction: multi-scan (**ABSCOR**; Higashi, 1995 ▶) *T*_min_ = 0.962, *T*_max_ = 0.975
7548 measured reflections
2464 independent reflections
1324 reflections with *I* > 2σ(*I*)
*R*_int_ = 0.114

#### Refinement

*R*[*F* ^2^ > 2σ(*F* ^2^)] = 0.063
*wR*(*F* ^2^) = 0.165
*S* = 1.06
2464 reflections
136 parameters
H-atom parameters constrained
Δρ_max_ = 0.31 e Å^−3^
Δρ_min_ = −0.30 e Å^−3^

### 

Data collection: *PROCESS-AUTO* (Rigaku, 1998 ▶); cell refinement: *PROCESS-AUTO*; data reduction: *CrystalStructure* (Rigaku/MSC, 2004 ▶); program(s) used to solve structure: *SIR97* (Altomare *et al.*, 1999 ▶); program(s) used to refine structure: *SHELXL97* (Sheldrick, 2008 ▶); molecular graphics: *Mercury* (Macrae *et al.*, 2006 ▶); software used to prepare material for publication: *publCIF* (Westrip, 2009 ▶).

## Supplementary Material

Crystal structure: contains datablocks I, global. DOI: 10.1107/S1600536809008769/is2397sup1.cif

Structure factors: contains datablocks I. DOI: 10.1107/S1600536809008769/is2397Isup2.hkl

Additional supplementary materials:  crystallographic information; 3D view; checkCIF report

## Figures and Tables

**Fig. 1.**
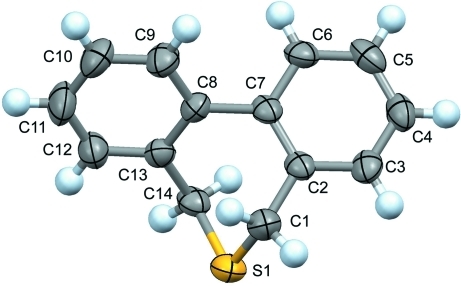
Perspective view of the title compound, (I), with the atom numbering scheme. Displacement ellipsoids are at the 70% probability level. H atoms are drawn as spheres of arbitrary radii.

**Fig. 2.**
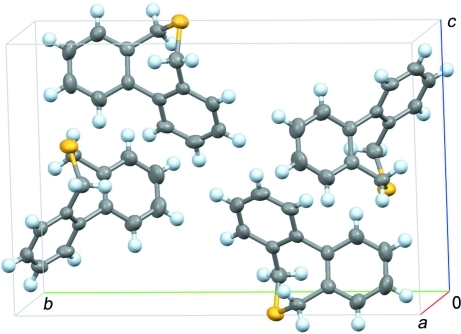
The crystal packing of title compound, (I). Displacement ellipsoids are at the 70% probability level. H atoms are drawn as spheres of arbitrary radii.

## supplementary crystallographic information

### Comment

As a part of our ongoing studies on the synthesis and structures of the transition metal complexes with thioether donor groups, we prepared a pair of atrop diastereomeric Rh^III^ complexes having a 2,2'-bis(2-aminoethylthiomethyl)-1,1'-biphenyl ligand (Yoshinari & Konno, 2008). We report herein the structure of the title compound, 5,7-dihydrodibenzo[*c*,*e*]thiepine, (I), which was accidentally obtained in the course of a direct synthesis of 2,2'-bis(2-aminoethylthiomethyl)-1,1'-biphenyl from 2,2'-bis(bromomethyl)-1,1'-biphenyl and 2-aminoethanethiol.

In the crystal structure of (I), two aromatic rings (C2—C7 and C8—C13) are inclined around the C7—C8 bond with a dihedral angle of 52.4 (1)°.

This compound (I) has been synthesized by treatment of 2,2'-bis(bromomethyl)-1,1'-biphenyl with sulfide anion, but has not been structurally characterized (Foubelo *et al.* 2005).

### Experimental

The reaction of 2,2'-bis(bromomethyl)-1,1'-biphenyl with 2-aminoethanethiol in ethanol gave a colorless solution. To the resulting solution was added diethylether, followed by allowing to stand in a refrigerator, which produced colorless stick crystals of (I) as a byproduct.

### Refinement

H atoms bonded to C atoms were placed at calculated positions (C—H = 0.95 or 0.99 Å) and refined as riding, with *U*_iso_(H) = 1.2*U*_eq_(C).

### Crystal data

**Table:**

C_14_H_12_S	*F*(000) = 448
*M**_r_* = 212.30	*D*_x_ = 1.267 Mg m^−^^3^
Monoclinic, *P*2_1_/*n*	Mo *K*α radiation, λ = 0.71075 Å
Hall symbol: -P 2yn	Cell parameters from 4537 reflections
*a* = 5.645 (3) Å	θ = 3.6–27.5°
*b* = 17.316 (9) Å	µ = 0.25 mm^−^^1^
*c* = 11.398 (5) Å	*T* = 200 K
β = 92.444 (19)°	Prismatic, colourless
*V* = 1113.1 (10) Å^3^	0.15 × 0.15 × 0.10 mm
*Z* = 4	

### Data collection

**Table:**

Rigaku R-AXIS RAPID diffractometer	2464 independent reflections
Radiation source: fine-focus sealed tube	1324 reflections with *I* > 2σ(*I*)
graphite	*R*_int_ = 0.114
Detector resolution: 10.00 pixels mm^-1^	θ_max_ = 27.5°, θ_min_ = 3.6°
ω scans	*h* = −4→7
Absorption correction: multi-scan (*ABSCOR*; Higashi, 1995)	*k* = −22→22
*T*_min_ = 0.962, *T*_max_ = 0.975	*l* = −14→14
7548 measured reflections	

### Refinement

**Table:**

Refinement on *F*^2^	Primary atom site location: structure-invariant direct methods
Least-squares matrix: full	Secondary atom site location: difference Fourier map
*R*[*F*^2^ > 2σ(*F*^2^)] = 0.063	Hydrogen site location: inferred from neighbouring sites
*wR*(*F*^2^) = 0.165	H-atom parameters constrained
*S* = 1.06	*w* = 1/[σ^2^(*F*_o_^2^) + (0.0655*P*)^2^] where *P* = (*F*_o_^2^ + 2*F*_c_^2^)/3
2464 reflections	(Δ/σ)_max_ < 0.001
136 parameters	Δρ_max_ = 0.31 e Å^−^^3^
0 restraints	Δρ_min_ = −0.30 e Å^−^^3^
0 constraints	

### Special details

**Table:**

Geometry. All e.s.d.'s (except the e.s.d. in the dihedral angle between two l.s. planes) are estimated using the full covariance matrix. The cell e.s.d.'s are taken into account individually in the estimation of e.s.d.'s in distances, angles and torsion angles; correlations between e.s.d.'s in cell parameters are only used when they are defined by crystal symmetry. An approximate (isotropic) treatment of cell e.s.d.'s is used for estimating e.s.d.'s involving l.s. planes.
Refinement. Refinement of *F*^2^ against ALL reflections. The weighted *R*-factor *wR* and goodness of fit *S* are based on *F*^2^, conventional *R*-factors *R* are based on *F*, with *F* set to zero for negative *F*^2^. The threshold expression of *F*^2^ > σ(*F*^2^) is used only for calculating *R*-factors(gt) *etc*. and is not relevant to the choice of reflections for refinement. *R*-factors based on *F*^2^ are statistically about twice as large as those based on *F*, and *R*- factors based on ALL data will be even larger.

### Fractional atomic coordinates and isotropic or equivalent isotropic displacement parameters (Å^2^)

**Table:**

	*x*	*y*	*z*	*U*_iso_*/*U*_eq_	
S1	1.27862 (17)	0.41043 (5)	−0.05701 (6)	0.0391 (3)	
C1	1.3831 (7)	0.39078 (19)	0.0932 (2)	0.0357 (8)	
H1	1.4103	0.3346	0.1023	0.043*	
H2	1.5369	0.4172	0.1083	0.043*	
C2	1.2131 (6)	0.41676 (17)	0.1826 (2)	0.0281 (7)	
C3	1.2565 (7)	0.48526 (17)	0.2447 (2)	0.0330 (8)	
H3	1.3926	0.5153	0.2294	0.040*	
C4	1.1020 (7)	0.50965 (19)	0.3284 (2)	0.0363 (9)	
H4	1.1289	0.5572	0.3684	0.044*	
C5	0.9098 (6)	0.4648 (2)	0.3532 (2)	0.0363 (9)	
H5	0.8061	0.4810	0.4120	0.044*	
C6	0.8660 (6)	0.39652 (19)	0.2937 (2)	0.0332 (8)	
H6	0.7331	0.3659	0.3123	0.040*	
C7	1.0160 (6)	0.37196 (17)	0.2059 (2)	0.0276 (8)	
C8	0.9612 (6)	0.30059 (17)	0.1387 (2)	0.0293 (7)	
C9	0.9182 (7)	0.23099 (19)	0.1957 (3)	0.0369 (8)	
H7	0.9236	0.2295	0.2790	0.044*	
C10	0.8682 (7)	0.1646 (2)	0.1335 (3)	0.0420 (9)	
H8	0.8406	0.1176	0.1738	0.050*	
C11	0.8582 (8)	0.1662 (2)	0.0120 (3)	0.0483 (10)	
H9	0.8242	0.1204	−0.0313	0.058*	
C12	0.8979 (7)	0.23477 (19)	−0.0461 (3)	0.0395 (9)	
H10	0.8892	0.2357	−0.1295	0.047*	
C13	0.9499 (6)	0.30201 (17)	0.0150 (2)	0.0270 (7)	
C14	0.9761 (6)	0.37741 (18)	−0.0492 (2)	0.0320 (8)	
H11	0.8833	0.4175	−0.0095	0.038*	
H12	0.9073	0.3716	−0.1300	0.038*	

### Atomic displacement parameters (Å^2^)

**Table:**

	*U*^11^	*U*^22^	*U*^33^	*U*^12^	*U*^13^	*U*^23^
S1	0.0500 (6)	0.0390 (5)	0.0295 (4)	−0.0065 (5)	0.0140 (4)	0.0003 (4)
C1	0.038 (2)	0.0342 (19)	0.0360 (16)	−0.0057 (17)	0.0081 (14)	−0.0011 (14)
C2	0.041 (2)	0.0229 (16)	0.0206 (13)	0.0035 (15)	0.0040 (12)	0.0033 (12)
C3	0.046 (2)	0.0246 (17)	0.0286 (15)	−0.0038 (16)	0.0003 (14)	0.0045 (13)
C4	0.057 (3)	0.0234 (17)	0.0289 (16)	0.0046 (17)	0.0016 (15)	−0.0023 (13)
C5	0.044 (2)	0.041 (2)	0.0239 (15)	0.0118 (18)	0.0062 (14)	−0.0004 (14)
C6	0.039 (2)	0.039 (2)	0.0226 (14)	−0.0031 (16)	0.0078 (13)	0.0036 (13)
C7	0.038 (2)	0.0242 (17)	0.0213 (14)	0.0017 (15)	0.0039 (13)	0.0045 (12)
C8	0.033 (2)	0.0237 (17)	0.0312 (15)	−0.0039 (15)	0.0029 (13)	0.0009 (12)
C9	0.045 (2)	0.0331 (19)	0.0326 (16)	−0.0082 (17)	−0.0004 (14)	0.0038 (14)
C10	0.048 (3)	0.0267 (19)	0.051 (2)	−0.0082 (18)	−0.0012 (17)	0.0045 (15)
C11	0.069 (3)	0.027 (2)	0.048 (2)	−0.009 (2)	−0.0008 (18)	−0.0093 (15)
C12	0.056 (3)	0.0317 (19)	0.0310 (16)	−0.0033 (18)	0.0023 (15)	−0.0054 (14)
C13	0.0226 (18)	0.0290 (18)	0.0296 (15)	−0.0006 (15)	0.0033 (12)	0.0012 (12)
C14	0.043 (2)	0.0316 (18)	0.0216 (14)	0.0011 (17)	0.0045 (13)	0.0035 (12)

### Geometric parameters (Å, °)

**Table:**

S1—C14	1.807 (4)	C7—C8	1.480 (4)
S1—C1	1.819 (3)	C8—C9	1.395 (4)
C1—C2	1.499 (4)	C8—C13	1.409 (4)
C1—H1	0.9900	C9—C10	1.374 (5)
C1—H2	0.9900	C9—H7	0.9500
C2—C7	1.391 (4)	C10—C11	1.384 (4)
C2—C3	1.397 (4)	C10—H8	0.9500
C3—C4	1.386 (5)	C11—C12	1.382 (5)
C3—H3	0.9500	C11—H9	0.9500
C4—C5	1.373 (5)	C12—C13	1.382 (4)
C4—H4	0.9500	C12—H10	0.9500
C5—C6	1.381 (4)	C13—C14	1.507 (4)
C5—H5	0.9500	C14—H11	0.9900
C6—C7	1.404 (4)	C14—H12	0.9900
C6—H6	0.9500		
			
C14—S1—C1	99.41 (15)	C9—C8—C13	118.6 (3)
C2—C1—S1	113.1 (3)	C9—C8—C7	121.2 (2)
C2—C1—H1	109.0	C13—C8—C7	120.2 (2)
S1—C1—H1	109.0	C10—C9—C8	121.3 (3)
C2—C1—H2	109.0	C10—C9—H7	119.3
S1—C1—H2	109.0	C8—C9—H7	119.3
H1—C1—H2	107.8	C9—C10—C11	119.8 (3)
C7—C2—C3	120.1 (3)	C9—C10—H8	120.1
C7—C2—C1	120.2 (3)	C11—C10—H8	120.1
C3—C2—C1	119.7 (3)	C12—C11—C10	119.8 (3)
C4—C3—C2	120.3 (3)	C12—C11—H9	120.1
C4—C3—H3	119.8	C10—C11—H9	120.1
C2—C3—H3	119.8	C13—C12—C11	121.2 (3)
C5—C4—C3	119.7 (3)	C13—C12—H10	119.4
C5—C4—H4	120.1	C11—C12—H10	119.4
C3—C4—H4	120.1	C12—C13—C8	119.3 (3)
C4—C5—C6	120.6 (3)	C12—C13—C14	120.6 (3)
C4—C5—H5	119.7	C8—C13—C14	120.0 (3)
C6—C5—H5	119.7	C13—C14—S1	114.2 (2)
C5—C6—C7	120.6 (3)	C13—C14—H11	108.7
C5—C6—H6	119.7	S1—C14—H11	108.7
C7—C6—H6	119.7	C13—C14—H12	108.7
C2—C7—C6	118.6 (3)	S1—C14—H12	108.7
C2—C7—C8	121.2 (3)	H11—C14—H12	107.6
C6—C7—C8	120.2 (3)		
			
C14—S1—C1—C2	−45.4 (3)	C2—C7—C8—C13	−51.6 (5)
S1—C1—C2—C7	79.6 (3)	C6—C7—C8—C13	127.2 (3)
S1—C1—C2—C3	−102.0 (3)	C13—C8—C9—C10	0.7 (5)
C7—C2—C3—C4	−1.0 (5)	C7—C8—C9—C10	−179.7 (4)
C1—C2—C3—C4	−179.4 (3)	C8—C9—C10—C11	−0.5 (5)
C2—C3—C4—C5	2.2 (5)	C9—C10—C11—C12	−0.2 (6)
C3—C4—C5—C6	−1.5 (5)	C10—C11—C12—C13	0.6 (6)
C4—C5—C6—C7	−0.5 (5)	C11—C12—C13—C8	−0.4 (6)
C3—C2—C7—C6	−1.0 (4)	C11—C12—C13—C14	−175.8 (4)
C1—C2—C7—C6	177.4 (3)	C9—C8—C13—C12	−0.3 (5)
C3—C2—C7—C8	177.8 (3)	C7—C8—C13—C12	−179.8 (3)
C1—C2—C7—C8	−3.7 (4)	C9—C8—C13—C14	175.1 (3)
C5—C6—C7—C2	1.8 (4)	C7—C8—C13—C14	−4.4 (5)
C5—C6—C7—C8	−177.1 (3)	C12—C13—C14—S1	−105.8 (3)
C2—C7—C8—C9	128.8 (3)	C8—C13—C14—S1	78.8 (3)
C6—C7—C8—C9	−52.3 (5)	C1—S1—C14—C13	−43.6 (2)
